# Radiomics models based on enhanced computed tomography to distinguish clear cell from non-clear cell renal cell carcinomas

**DOI:** 10.1038/s41598-021-93069-z

**Published:** 2021-07-02

**Authors:** Ping Wang, Xu Pei, Xiao-Ping Yin, Jia-Liang Ren, Yun Wang, Lu-Yao Ma, Xiao-Guang Du, Bu-Lang Gao

**Affiliations:** 1 CT/MRI Room, Key Laboratory of Cancer Radiotherapy and Chemotherapy Mechanism and Regulations, Affiliated Hospital of Hebei University, 212 East Yuhua Road, Baoding, 071000 Hebei Province China; 2 GE HealthCare, ShangHai, 200120 China; 3 Chengde Medical University, Chengde, 067000 Hebei Province China

**Keywords:** Computational biology and bioinformatics, Biomarkers, Medical research, Oncology

## Abstract

This study was to assess the effect of the predictive model for distinguishing clear cell RCC (ccRCC) from non-clear cell RCC (non-ccRCC) by establishing predictive radiomic models based on enhanced-computed tomography (CT) images of renal cell carcinoma (RCC). A total of 190 cases with RCC confirmed by pathology were retrospectively analyzed, with the patients being randomly divided into two groups, including the training set and testing set according to the ratio of 7:3. A total of 396 radiomic features were computationally obtained and analyzed with the Correlation between features, Univariate Logistics and Multivariate Logistics. Finally, 4 features were selected, and three machine models (Random Forest (RF), Support Vector Machine (SVM) and Logistic Regression (LR)) were established to discriminate RCC subtypes. The radiomics performance was compared with that of radiologist diagnosis. In the testing set, the RF model had an area under the curve (AUC) value of 0.909, a sensitivity of 0.956, and a specificity of 0.538. The SVM model had an AUC value of 0.841, a sensitivity of 1.0, and a specificity of 0.231, in the testing set. The LR model had an AUC value of 0.906, a sensitivity of 0.956, and a specificity of 0.692, in the testing set. The sensitivity and specificity of radiologist diagnosis to differentiate ccRCC from non-ccRCC were 0.850 and 0.581, respectively, with the AUC value of the radiologist diagnosis as 0.69. In conclusion, radiomics models based on CT imaging data show promise for augmenting radiological diagnosis in renal cancer, especially for differentiating ccRCC from non-ccRCC.

## Introduction

Renal cell carcinoma (RCC) is a kidney cancer that originates in the lining of the proximal convoluted tubule, a part of very small tubes in the kidney that transport primary urine^1^. RCC accounts for 2–3% of adult malignant tumors worldwide and has a high mortality rate among urinary system tumors. Pathological classification of RCCs includes clear cell RCC (ccRCC) and non-clear cell RCC (non-ccRCC). The non-ccRCC involves chromophobe (chRCC), papillary RCC (pRCC), collecting duct carcinoma (CDC) and unclassified RCC, with the incidence of ccRCC, pRCC, chRCC and CDC of about 70%, 10–20%, 5–10% and 1%, respectively. Unclassified RCC has rarely been reported^1–3^.

The diagnosis of RCC mainly relies on enhanced computed tomography (CT) scanning of the kidney, however, a high misdiagnosis rate still exists in clinical practice even though different distinguishable enhancement modes exist for ccRCC and non-ccRCC. Use of different kinds of images (CT, magnetic resonance imaging, ultrasound, etc.) in combination may help improve the diagnostic accuracy for different types of RCC, however, to obtain these images may increase the patients’ financial burden, radiation dosage, and workload for the physician, thus making a timely diagnosis impossible. Moreover, no specific tumor markers exist for different types of RCC. The currently most important prognostic factor for RCC is pathological staging^4^. Previous studies reported a high incidence of metastasis for ccRCC, and ccRCC and non-ccRCC have different responses to molecular target therapy, especially in advanced metastatic RCC^5,6^. Moreover, the targeted therapies for ccRCC and non-ccRCC and their prognoses are quite different^7^, and the prognosis of non-ccRCC is better than that of ccRCC. Therefore, early diagnosis of RCC subtypes is the key to good effects of clinical treatment^7,8^. Although renal biopsy is the gold standard for correct diagnosis, it can cause various unpredictable risks, such as metastasis of cancer cells, hemorrhage of tumors, and sampling errors^9–11^.

In 2012, Lambin et al.^12^ first defined the concept of radiomics by extracting more information (texture, shape, intensity, high-order features, etc.) from medical images using advanced feature analysis before converting the features into collectible radiomics data to diagnose the nature of lesions and help radiologists make correct decision. Although radiomic features cannot be observed with human eyes, several cases have shown that these features are closely associated with the phenotype of cancer and the microenvironment of cancer cells^13–15^. Studies have also demonstrated that these features are highly reliable in distinguishing tumor types and predicting survival in different types of cancer^13,15^. In addition, image texture analysis can be used to distinguish benign from malignant tumors as well as the invasiveness of tumors, such as non-small cell lung carcinoma (NSCLC) and liver cancer^16^. The gray level co-occurrence matrix has been applied to distinguish benign from malignant breast cancers with the accuracy of about 90%^17,18^.

Review of the literature on discrimination of RCC subtypes identified only one article establishing the radiomics models of Boruta and mRMRe combined with von Hippel-Lindau (VHL) gene mutation to distinguish ccRCC from non-ccRCC, with good performance^19^. This study developed the radiomics model with all-relevant imaging features based on multiphasic CT scanning images to investigate the possible radiogenomics link between the imaging features and a key ccRCC driver gene—the von Hippel-Lindau (VHL) gene mutation. A meta-analysis of radiomics studies in differentiating angiomyolipoma without visible fat from RCC demonstrated moderate diagnostic odds ratios of 6.24 (95% CI 4.27–9.12; p < 0.001) and moderate methodological diversity^20^. These radiomics programs have displayed a promising future in solving clinical problems where subjective interpretation is not established or challenging. Traditional enhanced CT imaging data cannot achieve better diagnostic performance and requires joint diagnoses, whereas imaging radiomics can have specific characteristics to achieve results that cannot be matched by traditional medical imaging data. We hypothesized that some models of radiomics might be established to better differentiate ccRCC from non-ccRCC so as to improve the diagnosis accuracy, and this study served this purpose.

## Results

### Patients’ characteristics

A total of 208 patients with 208 cancers were identified. After exclusion of 18 patients, 190 patients were enrolled in this study, including 147 cases with ccRCC and 43 cases with non-ccRCC (24 cases with pRCC, 13 chRCC and 6 CDC) (Table 1). There were 100 males and 90 females, with an age range of 27–88 years (mean 59.32 ± 10.68 years). In postoperative pathological findings, the size of cancer ranged 2–11 cm (mean 6.5 ± 3.5 cm). All these 190 patients were divided into the training set and the testing set according to the ratio of 7:3 (Table 1). The training set had 132 patients including 68 males and 64 females with an age range of 27–86 years (mean 58.2 ± 9.9), whereas the testing set had 58 patients comprising 32 males and 26 females with an age range of 30–88 years (mean 60.2 ± 9.1). The distribution of RCC was ccRCC in 102 cases and non-ccRCC in 30 cases (pRCC in 17 cases, chRCC in 9 and CDC in 4) in the training set and ccRCC in 45 cases and non-ccRCC in 13 cases (pRCC in 7 cases, chRCC in 4 and CDC in 2) in the testing set. No significant (P > 0.05) difference existed in the age, sex and disease distribution between the groups of patients.

**Table 1:** Demographic information in two sets.

Variables	Training set	Testing set
Cases	132 (70%)	58 (30%)
**Sex**		
Male	68 (51.5%)	32 (55.2%)
Female	64 (48.5%)	26 (44.8%)
Age, mean (range, y)	58.2 (27–86)	60.2 (30–88)
≤ 60 (y)	60 (45.5%)	25 (43.1%)
> 60 (y)	72 (54.5%)	33 (56.9%)
**Subtype**		
ccRCC	102 (77.3%)	45 (77.6%)
Non-cc RCC	30 (22.7%)	13 (22.4%)
pRCC	17	7
chRCC	9	4
CDC	4	2

*ccRCC* clear cell renal cell carcinoma, *pRCC* papillary RCC, *chRCC* chromophobe RCC, *CDC* collecting duct carcinoma.

### Inter-observer and intra-observer reproducibility of Radiomics feature extraction

The mean ICC value of radiomics feature was 0.78 indicating good consistency in inter- and intra-observer reproducibility of Radiomics feature extraction.

### Predictive performance of models

**Figure 1:**
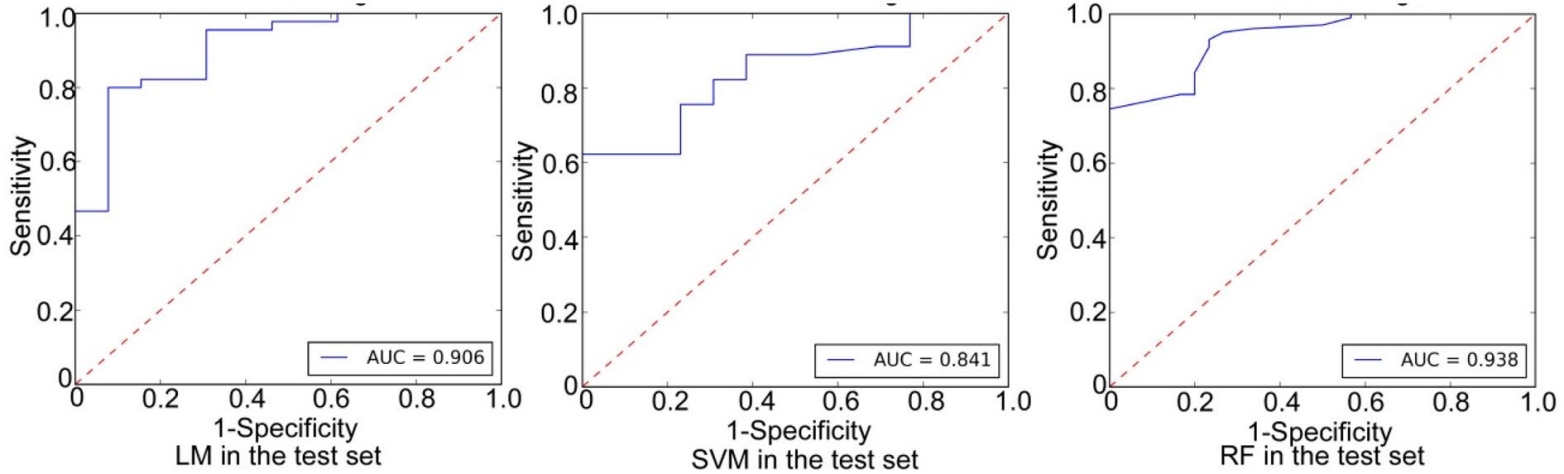
Receiver operating characteristic (ROC) curve analysis of three radiomics models for differentiating clear cell renal cell carcinoma (RCC) from papillary RCC and chromophobe RCC in the testing set. *LM* logistic regression model, *SVM* support vector machine model, *RF* random forest model.

The ROC curve analysis demonstrated that in the diagnosis of ccRCC in the testing set (Table 2 and Fig. 1), the RF model had an AUC value of 0.909, a sensitivity of 0.956, and a specificity of 0.538. The SVM model had an AUC value of 0.841, a sensitivity of 1.0 and a specificity of 0.231. The LR model had an AUC value of 0.906, a sensitivity of 0.956, and a specificity of 0.692.

**Table 2:** Parameters of ccRCC and non-ccRCC in the testing set.

Model	AUC	Specificity	Sensitivity	F1	PP	NP
RF	0.909	0.538	0.956	0.915	0.878	0.778
LR	0.906	0.692	0.956	0.935	0.915	0.818
SVM	0.841	0.231	1.0	0.9	0.818	1.0

*ccRCC* clear cell renal carcinoma, *AUC* area under the curve, *PP* positive prediction, *NP* negative prediction, *RF* random forest model, *LR* logistic regression model, *SVM* support vector machine. PP = ccRCC, and NP = non-ccRCC.

**Figure 2:**
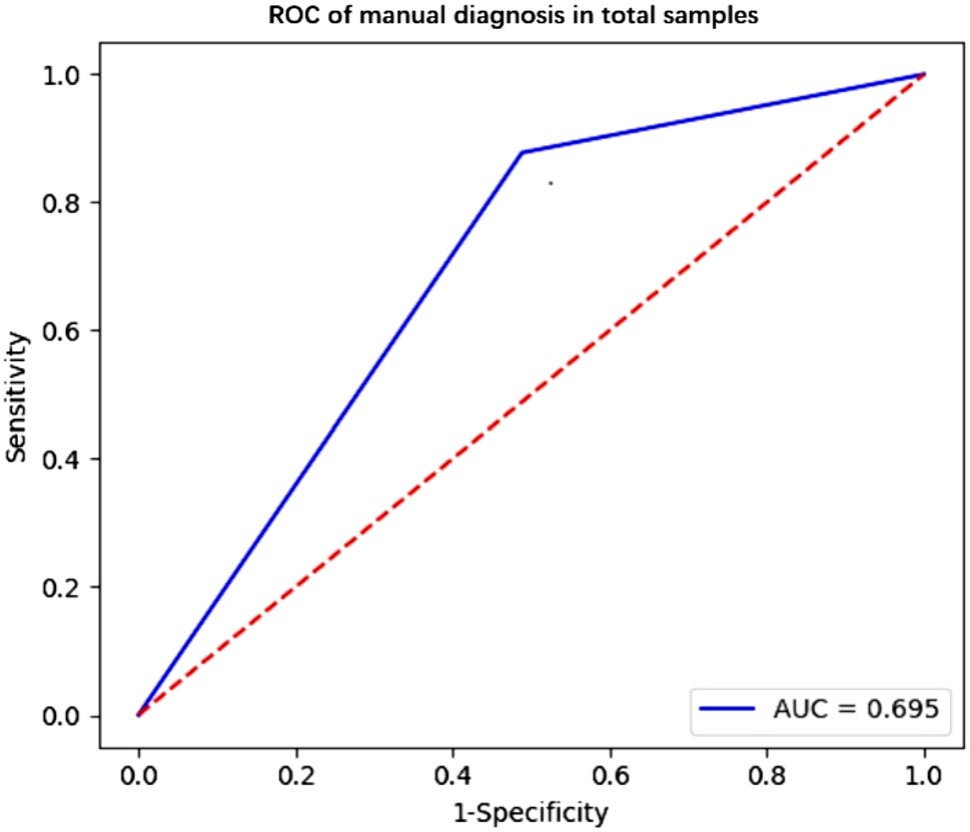
Receiver operating characteristic (ROC) curve analysis of radiologist diagnosis in the total samples, with the AUC of 0.69, sensitivity of 0.85, and specificity of 0.581.

In radiologist diagnosis based on the contrast-enhanced CT imaging data in comparison with the pathological findings, the sensitivity in diagnosing ccRCC was 85.0% (125/147), the specificity was 58.1% (25/43), and the AUC value was 0.69 (Table 3, and Fig. 2).

**Table 3:** Radiologist diagnosis of ccRCC and non-ccRCC compared with pathological findings (n = 190).

Radiologist diagnosis	Pathological diagnosis		Total
	ccRCC (n = 147)	Non-ccRCC (n = 43)	
ccRCC	125	18	143
Non-ccRCC	22	25	47
Total	147	43	190

*ccRCC* clear cell renal carcinoma.

## Discussion

In this study, three radiomic models (RF model, SVM model and LR model) were established to differentiate ccRCC from non-ccRCC, and it was demonstrated that the LR model is the best among the three models with an AUC value of 0.906, a sensitivity of 0.956, and a specificity of 0.692 in the testing set. In terms of sensitivity and specificity, the radiomics results were better than those achieved by radiologist diagnosis (sensitivity = 0.85, specificity = 0.581, and AUC = 0.69). The radiomics results are better than that achieved in the study by Andreas et al.^21^ who had developed a quantitative multiparametric MRI approach to differentiate ccRCC from other renal cortical tumors (AUC = 0.804). Ordinary imaging diagnosis plays poorly than the results of artificial intelligence using imaging radiomics. The performance of radiomics models in our study was also better than the performance of tumor biomarker of GPX1 which has an AUC of 0.7908 (95% CI 0.7409–0.8407) to different ccRCC from normal tissue^22^.

RCC is the most common malignant tumor of the kidney, accounting for about 4% of all new tumors with a high metastasis rate of 20%, and early diagnosis of RCC plays a pivotal role in clinical guidance and treatment^7,8^. Although multi-phase contrast-enhanced CT can be used to distinguish different subtypes of RCC, there is still a high misdiagnosis rate in clinical practice^8,23^. Currently, few studies have utilized radiomic features to assess and distinguish RCC subtypes^24^, and other studies have assessed mode^8^, iodine quantification^23^, two-dimensional (2D) texture^9–11^ of enhanced CT scan and apparent diffusion coefficient^25^, in addition to other quantitative parameters of magnetic resonance (MR) images^26^. The radiomics model has been favored by some scholars because of its high reproducibility.

Tumor blood vessels are more abundant at the tumor–host interface than in central regions of the organ. Zhu et al.^27^ measured the most obvious enhancement in tumors and reported that low enhancement is the main characteristic of high-grade ccRCC. In the present study, VOI was drawn in renal tumors in the cortical phase. The image features of tumors (e.g., gray, wavelet features, and texture features) can reflect the heterogeneity of tumors. Heterogeneity, as one of the important characteristics of malignant tumors, is associated with biological behavior of human malignant tumors^28^. It can reflect changes in growth-related factors like the vascular endothelial growth factor (VEGF) and changes of tumor microenvironment, which can cause cell proliferation or apoptosis, promotion or inhibition of metabolic activity, and increase or decrease of angiogenesis in the tumors^29^. Heterogeneity of tumor angiogenesis may lead to reduction of local effective blood flow, hypoxia, increased interstitial hydrostatic pressure in the tumor microenvironment and finally increased risk of tumor invasiveness and metastasis. Necrosis in the tumor on enhanced CT scan has been found to be an independent predictor of biological invasive ccRCC^30,31^. Shun et al.^32^ attempted to discriminate low grade (Fuhrman I/II) and high grade (Fuhrman III/IV) ccRCC by using CT-based radiomic features and suggested that radiomic features could be used as biomarkers for the preoperative evaluation of the ccRCC Fuhrman grades.

Based on the 3D structures of tumors, our study explored the heterogeneity among different RCC subtypes and revealed differences between different RCC subtypes in the cortical phase of enhancement. We extracted 396 features and final four stable parameters for radiomics modeling. The LR model was demonstrated to improve the ability to distinguish subtypes of RCC, with higher AUC value (0.909) in the testing set. Besides, LR is an ensemble method, which can predict the linear data more stably and process a huge amount of data. Ding et al.^33^ achieved satisfactory results in the study of texture-based scoring for distinguishing high and low grades of RCC with an AUC value of 0.878.

Previous studies on imaging radiomics were mainly focused on 2D texture analysis of renal tumors, while 3D texture analysis was rarely undertaken. Three-D analysis is more comprehensive and reliable to better understand the heterogeneity of tumors and deeply reveal the imaging characteristics of tumors. Our study analyzed the images with 3D-VOI method, extracted different radiomic features and developed radiomics models to distinguish ccRCC from non-ccRCC with good performance. Class imbalance is a common problem which has been studied by machine learning and data mining researchers. It can happen when the instance of one class outnumbers the instances of other classes. Feature selection techniques, classifying parameters, and data endpoints may all significantly affect the predictive accuracy in radiomics-based predictive models for cancer prognosis^34^. The LR model achieved the best AUC, specificity, F1 score, and positive prediction value (Table 2). The sensitivity of LR model is lower than the other models, which may be caused by the imbalance of data. The LR model performance was more stable, which was mainly reflected by the highest specificity. In a study, the SMOTE method was used to expand the sample with a small number of categories^35^. However, this method may generate some fake data, and its effectiveness could not be guaranteed^36^.

The specificities of the models in our study were generally low. Specificity can be understood as sensitivity to negative samples. In a task with a small number of negative samples, the specificity of the classifier is manifested in its ability to make specific and special judgments when the population of negative samples is small. The higher the specificity of the detection method, the more effective the detection method. In this case, the higher the specificity of the radiomics models, the more effective to differentiate ccRCC from non-ccRCC. Compared with tumor markers and other imaging tests^21,22^, the sensitivity and specificity in our study were better.

Some limitations may exist in this study including a relatively small number of patients, Chinese ethnicity only, retrospective nature in the study, limited number of non-ccRCC and single center study, which might reduce the effect of this study. The high sensitivity and low specificity of this study were largely related to the unbalanced distribution of the screened cases and the small number of non-ccRCCs. Because of the low incidence of non-ccRCC, the number of cases was small in our study.

In conclusion, radiomics models show promise for augmenting radiological diagnosis in renal cancer, especially for differentiating ccRCC from non-ccRCC. The differentiation of ccRCC and non-ccRCC has been investigated repeatedly, and radiomics analysis may have moderate ability of differentiation. However, well-designed and appropriately powered prospective radiomics trials using the novel imaging markers may be able to demonstrate a promising future in diagnosis of renal cancers.

## Patients and methods

### Patients

This retrospective study was approved by the Institutional Review Committee of the Affiliated Hospital of Hebei University (Baoding, China) with all patients given their signed informed consent to participate. All methods were performed in accordance with the relevant guidelines and regulations. From January 1, 2017 to December 20, 2018, patients who had RCC with plain and enhanced CT scanning of the kidney in our hospital were reviewed. Inclusion criteria were patients with postoperative RCC confirmed by pathology including ccRCC and non-ccRCC (stage T1–T4) and with preoperative enhanced-CT scan. Exclusion criteria were patients with necrosis and cystic degeneration over 4–5 cm in the cancer lesion, metastatic RCC, and unsatisfactory quality of imaging due to motion artifacts or difference in contrast medium injection. The patients were randomly divided into the training set and the testing set according to the ratio of 7:3.

### CT images acquisition

Examination was performed with renal plain and enhancement CT scan with a 64-row scanner (GE Discovery HD 750; GE Health Care, Chicago, IL, USA), with all patients fasting for 6 to 8 h before scanning. The scanning sequences and the related parameters were as follows: for cortical phase, medullary phase, and excretion phase, the duration of scanning was 30–35, 50–60, and 180 s after the injection of contrast agent, respectively. Iodophor alcohol (Jiangsu Hengrui Medicine Co., Ltd., Nanjing, China), a non-ionic iodine contrast agent, was injected through the elbow vein at a rate of 3.0–3.5 ml/s and the total injection amount was 0.90–1.01 ml/kg according to the patient’s body weight. Layer thickness was 5 mm, pitch 0.984:1, scanning field 36 cm × 43 cm, matrix 512 × 512, tube voltage 100–120 kV, tube current 134–409 mA, window width 250 ~ 450 HU, and window level 30 -50 HU.

### Image processing

**Figure 3:**
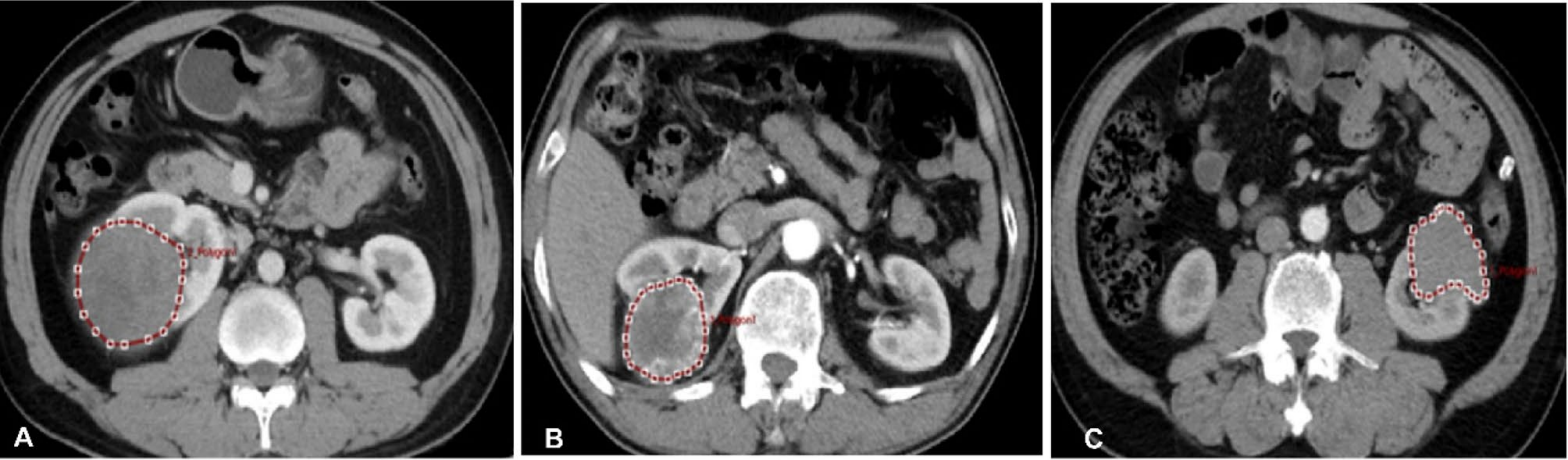
Renal cell carcinoma. (**A**) A 59-year-old man had cortical phase of papillary renal cell carcinoma (RCC) on computed tomography (CT) enhancement (ROI with dotted line). (**B**) A 62-year-old woman had cortical phase of clear cell RCC on CT enhancement (ROI with dotted line). (**C**) A 71-year-old man had cortical phase of chromophobe RCC on CT enhancement (ROI with dotted line).

All CT images were resampled into isotropic voxel size (1 mm × 1 mm × 1 mm). The enhanced-CT scan images of 190 cases (in the cortical phase) were loaded into the ITK-SNAP software. The region of interest (ROI) was segmented by two diagnostic physicians with 15–20 years of working experience. To verify the consistency of ROI against the intra- and inter-observer delineation variations, the delineation was repeated on 40 patients (20 patients with ccRCC and 20 with non-RCC who were randomly chosen) by the same radiologist (Reader 1 with 15 years’ experience in urologic imaging) for intra-observer assessment and by the other radiologist (Reader 2 with 20 years’ experience in urologic imaging) for interobserver assessment. When the intraclass correlation coefficient (ICC) was greater than 0.75, it was considered in good agreement, and the remaining image segmentation was performed by reader 1. The delineation principles of ROI (Fig. 3) were 0–1 mm in the medial margin in the cortical phase was selected for delineation with the ROI being delineated layer-by-layer to obtain the volume of the interest (VOI).

### Feature selection

**Figure 4:**
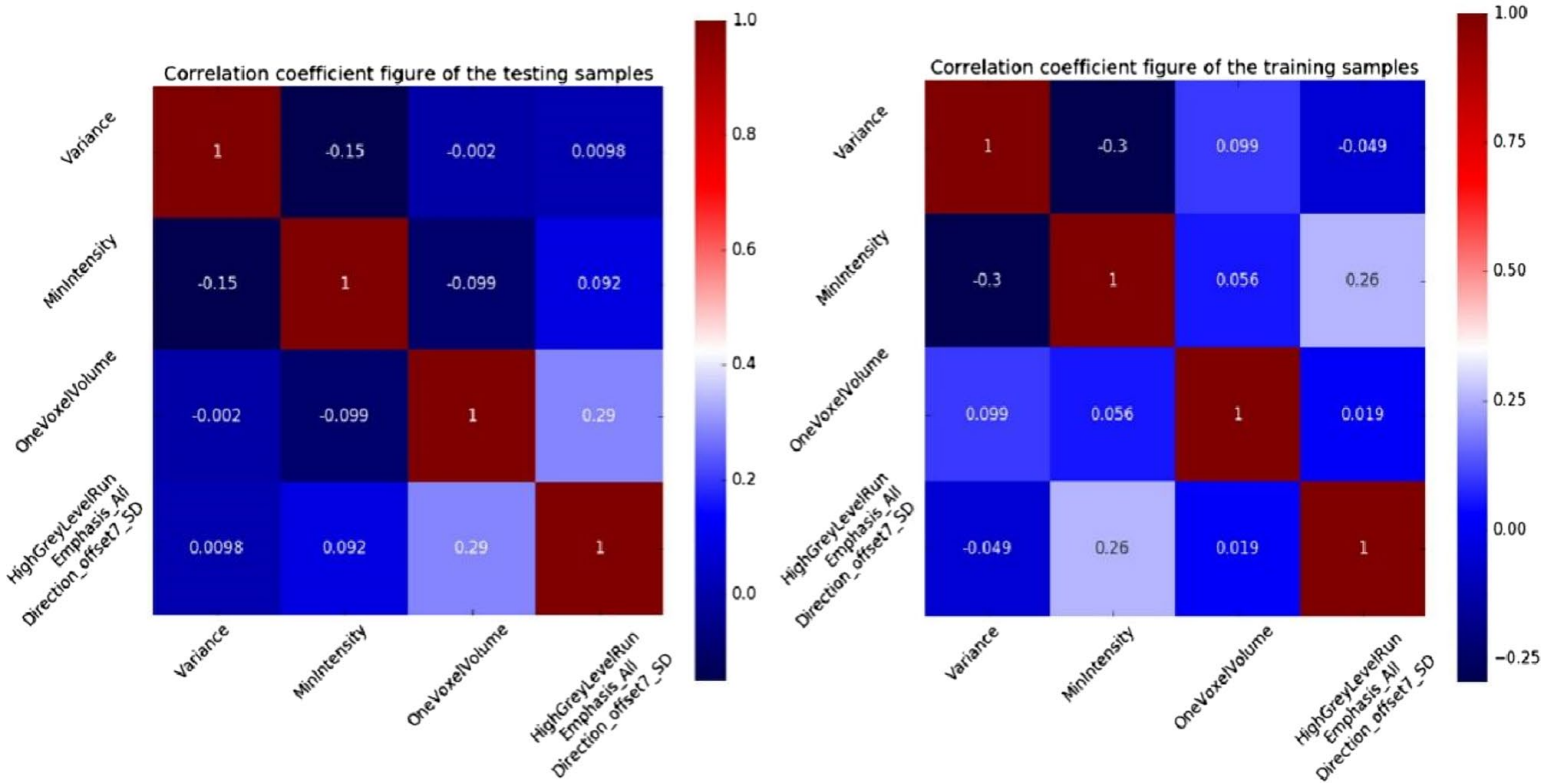
Correlation coefficients between four features in the samples in the training and test sets. The coefficient factors between the four features were all < 0.3, indicating no linear relationship, and could be used as independent predictive factor.

All original image files and delineated ROI files were imported into the Artificial Intelligence Kit software (Artificial Intelligence Kit V3.0.0.R, GE Healthcare) for feature extraction. Before feature extraction, the images were discretized with a bandwidth set 25. Radiomics features were extracted including Histogram, Haralick, Formfactor, Gray level size zone matrix (GLSZM), Gray-level co-occurrence matrix (GLCM) and Run-length matrix (RLM). In total, 396 imaging features were extracted in each patient before feature selection, including 42 Histogram features, 10 Haralick, 9 Formfactor features, 11 GLSZM, 144 GLCM features and 180 RLM features. Extracted texture features were standardized to remove the unit limits of the data of each feature. The dimension reduction was then performed using the ANOVA test, general linear model and the first 10% mutual information to select the features with statistical significance including LargeAreaEmphasis, OneVoxelVolume, HaralickCorrelation_angle0_offset7, ClusterProminence_angle35_offset7, ShortRunEmphasis_angle0_offset4, LowGreyLevelRunEmphasis_AllDirection_offset1_SD, HighGreyLevelRunEmphasis_AllDirection_offset7_SD, HaralickCorrelation_angle35_offset4, LeastAxisLegnth, LowIntensityLargeAreaEmphasis, MaxIntensity, MinIntensity, HistogramEntropy, Variance, HistogramEnergy, MeanValue, SedDeviation and HaraEntroy. The correlation between features, Univariate Logistic and Multi-Variate Logistic was firstly analyzed, and then, the correlation test was calculated to reduce data redundancy before four relatively stable features were selected, including Variance, HighGreyLevelRunEmphasis_AllDirection_offset7_SD, MinIntensity and OneVoxelVolume (Fig. 4).

### Machine learning models

A Logistic regression (LR) model, a Random forest (RF) model and a Support vector machine (SVM) model were built from the established stable optimal feature subsets in the training set and tested in the testing set. The hyper-parameters of the SVM model were automatically selected by the grid search method. The kernel, gamma and C were “rbf”, 0.1 and 0.1, respectively. In this study, the grid search method was used to tune the machine learning models. No hyperparameters were used to tune the LR model, the RF was tuned with 100 trees, and the SVM was tuned with C set as 0.1. The ccRCC data were set as the positive class while the non-ccRCC as the negative class. The performance of the machine learning models was compared with that by radiologist diagnosis, which was made by two senior radiologists based on the imaging data in comparison with pathological findings.

### Statistical analysis

All statistical analyses for the present study were performed with R 3.5.1 (www.rproject.org) and Python 3.5.6 (www.python.org). The independent t test was used to compare the difference between the training and testing sets. Receiver operating characteristic (ROC) curve was performed to determine the performance of the machine learning models in distinguishing ccRCC and non-ccRCC. The sensitivity, specificity, positive prediction, negative prediction, and area under curve (AUC) were calculated. A two-tailed p-value < 0.05 indicated statistical significance.
